# Using Discarded Facial Tissues to Monitor and Diagnose Viral Respiratory Infections

**DOI:** 10.3201/eid2903.221416

**Published:** 2023-03

**Authors:** Gisele Lagathu, Claire Grolhier, Juliette Besombes, Anne Maillard, Pauline Comacle, Charlotte Pronier, Vincent Thibault

**Affiliations:** Centre Hospitalier Universitaire Rennes, Rennes, France (G. Lagathu, C. Grolhier, J. Besombes, A. Maillard, P. Comacle, C. Pronier, V. Thibault);; University of Rennes, Rennes (C. Grolhier, J. Besombes, C. Pronier, V. Thibault)

**Keywords:** viral respiratory infections, diagnosis, upper respiratory tract infection, matrices, epidemiology, surveillance, viral ecology, viruses, respiratory infections, zoonoses

## Abstract

Molecular biology amplification enables sensitive detection of most respiratory viruses through nasopharyngeal swabbing. We developed an innovative approach to detect viral genomes on used facial tissues. In 2 communities of children, used tissues were collected once weekly for 1 year. Pooled analysis of tissues enabled detection of successive virus circulation in 4 age groups over time and forecasted by several weeks the circulation of influenza in the general population. At the individual level, in a proof-of-concept study of 30 volunteers with influenza-like signs/symptoms, we identified common respiratory viruses. The signals for SARS-CoV-2 obtained in parallel from 15 facial tissues and swab samples were similar and often higher for the tissues (11/15). Individual analysis of tissues offers a noninvasive, sensitive, and affordable alternative to self-sampling without a medical care requirement. Pooled analyses may be used to detect virus spread in specific communities, predict seasonal epidemics, and alert the population to viral infections.

Respiratory viral infections (RVIs) are the most common virus-induced pathologies. Associated signs/symptoms range from common colds with simple rhinorrhea without fever to acute respiratory distress syndrome sometimes leading to death (*1*). In addition to their clinical effects, RVIs have a major economic effect because of not only healthcare resource use (direct costs) but also productivity losses (indirect costs), and they paradoxically receive little attention (*2*,*3*). Moreover, antimicrobial treatments are too often prescribed for RVIs, thereby disseminating antimicrobial resistance.

Respiratory viruses can be detected all year, but they seem to circulate more during winter, when temperature, humidity, and behavioral patterns are favorable for their dissemination (*4*). Although this observation holds true for classical winter RVIs, our recent experience with SARS-CoV-2 and unprecedented observations of several worldwide epidemic waves of COVID-19 independent of the season should also be mentioned. Respiratory virus year-round circulation follows multicomponent rules, still imperfectly understood (*5*). RVIs cause mostly upper respiratory illnesses, particularly in adults, but are also associated with severe lower respiratory tract signs/symptoms, causing a major disease burden among children (*6*).

In recent years, diagnosis of viral infection has largely benefited from progress in molecular biology methods. Several easy-to-perform commercial assays are available, and switching from manual techniques requiring a high level of expertise to highly automated approaches has made simultaneous detection of most common respiratory viruses much easier (*7*). Syndromic panels also offer rapid diagnosis, enabling adapted care for the most severely ill patients (*8*,*9*). As it becomes easier to provide an accurate diagnosis, correct identification of pathogens in RVIs deserves more attention. Associating a virus with a disease, even a simple cold, has several advantages. For children, diagnosing a viral cause of respiratory infection may limit the use of antimicrobial therapy, reassure the parents, and avoid further transmission to the community or even nosocomial infection (*10*,*11*). For adults, diagnosis could help control transmission, particularly to the most vulnerable populations; SARS-CoV-2 is a good example. The main drawback lies in the cost of diagnosing respiratory viruses, but costs may go down in the future.

Detecting respiratory viruses is hampered by the collection method, relying on nasal swab sampling that is usually considered invasive by patients (*12*). Although nasopharyngeal swab sample testing is considered the most sensitive, alternative approaches have been proposed, particularly for COVID-19 diagnosis. Saliva or oropharyngeal swab samples seem to be reliable in terms of sensitivity, but they have not been extensively tested for all common respiratory viruses. Blaschke et al. have proposed testing used facial tissues to diagnose RVIs in children (*13*). They analyzed only a small surface of the tissue yet obtained relative satisfactory sensitivity (up to 84%). The most sensitive sample type overall was nasal aspirate, but facial tissue tended to be more sensitive for older children.

With this study, we aimed to determine the potential of used facial tissue for documenting RVIs in different settings. We collected data as a population approach after pooling used tissues from different communities of children and as an individual approach among adults or children. All participants either already had a diagnosis of RVI (positive controls) based on a standard nasal swab sample tested by using the same diagnostic assay or had respiratory signs/symptoms consistent with an upper respiratory tract viral infection.

All participants were informed about the purpose of the study. Because nose blowing is an individual act, participation was intentional and voluntary. Most volunteers were either family members, professionals working in the laboratory, or their relatives. This type of research is in agreement with the Jardé law on biomedical research and does not require additional authorization. Nevertheless, we obtained approval from the ethics committee of the University of Rennes.

## Material and Methods

### Community Testing

We conducted the pooled collection study in 2 parts. The first part was conducted in a daycare center located within the Centre Hospitalier Universitaire (Rennes, France), which hosted mostly employees’ children. Collection of used facial tissues started on week 40 of 2018 and continued through week 15 of 2019. The second part was conducted in a preschool (*maternelle)*, from week 37 of 2019 through week 10 of 2020. For both locations, parents were informed of the study, and results were regularly communicated by the directors on an informal basis.

In the first part of the study, at the daycare center all used tissues were collected anonymously each week and pooled. The daycare facility could accommodate up to 40 children in each of 2 units, divided by age: youngest (<19 months of age) and oldest (19 months–4 years of age). The daycare staff collected all tissues during the week, and the container was collected each Friday; tissues were used for either nose blowing or, for the younger children, nose washing with a small amount of physiologic solution. For the second part of the study, the preschool comprised 2 classes of 20 pupils each: the first (3 years of age) and the second (4 years of age) grades. Tissues were used only for nose blowing, and the children were asked to throw their tissues in the specific container instead of the garbage. Each Friday, the filled containers were collected and clean empty containers were delivered for the following week.

### Individual Collection of Tissue

For 15 volunteers with COVID-19 diagnosed by reverse transcription PCR of a standard nasopharyngeal swab sample, results obtained the same day on individual facial tissues enabled comparison of cycle threshold (Ct) values between both matrices. To enable documentation of secretion kinetics over 5 days, 2 volunteers, after COVID-19 diagnosis, agreed to provide daily nose discharge from nasopharyngeal swab samples.

### Processing of Collected Material

To determine the volume of soaking buffer needed, each week, we counted all tissues from each container. Eventually, we chose a volume of 7 mL of Dulbecco’s phosphate-buffered saline (DPBS; GIBCO, https://www.thermofisher.com) per tissue. We collected the saturated liquid after the pooled tissues had been gently soaked for few minutes and then pressed with a home-brew device. For individual collections, we introduced the used tissues into a 60-mL syringe soaked with 7 mL of DPBS. We then firmly pressed the syringe plunger onto the soaked tissue and, depending on the tissue absorbing capacity, regularly obtained 5 mL of residual liquid.

### Viral Genome Detection

We used commercial kits to detect viral genomes. For pooled collections, we used Allplex Seegene (Eurobio, https://www.eurobio-scientific.com) on the sample of collected liquid, according to the manufacturer’s recommendations. The kit detects influenza A virus; influenza A(H1) virus; influenza A(H1N1)pdm09 virus; influenza A(H3) virus; influenza B virus; respiratory syncytial viruses A and B; adenovirus; enterovirus; human metapneumovirus (hMPV); parainfluenza viruses 1–4; bocaviruses 1–4; coronaviruses 229E, NL63, and OC43; and human rhinovirus. For individual testing, and particularly to detect SARS-CoV-2 RNA, we used TaqPath (ThermoFisher Scientific, https://www.thermofisher.com), Alinity (Abbott, https://www.corelaboratory.abbott), GeneXpert (Cepheid, https://info.cepheid.com), and FilmArray (bioMérieux, https://www.biomerieux.com). To detect cytomegalovirus (CMV), Epstein-Barr virus, and human parvovirus B19 genome, as part of our routine daily workflow we used RealStar assays (Altona, https://www.altona-diagnostics.com). All products were used as recommended by the manufacturers. For the kinetic study on SARS-CoV-2–positive samples and the comparison of signals between nasopharyngeal swab samples and tissues, we obtained all Ct values from a SARS-CoV-2 AMP Kit automatized on Alinity. This test amplifies 2 genome target genes (RNA polymerase and nucleocapsid), and the obtained signal is provided as a single Ct. Concordance between the signals obtained by the different commercial techniques routinely used in our laboratory is excellent. Our laboratory is certified according to COFRAC NF EN ISO 15189.

### Statistical Analyses

To compare Ct values obtained from nasopharyngeal swab and tissue samples, we used a Wilcoxon test. We considered p<0.05 to be significant.

## Results

### Pooled Material

**Figure 1:**
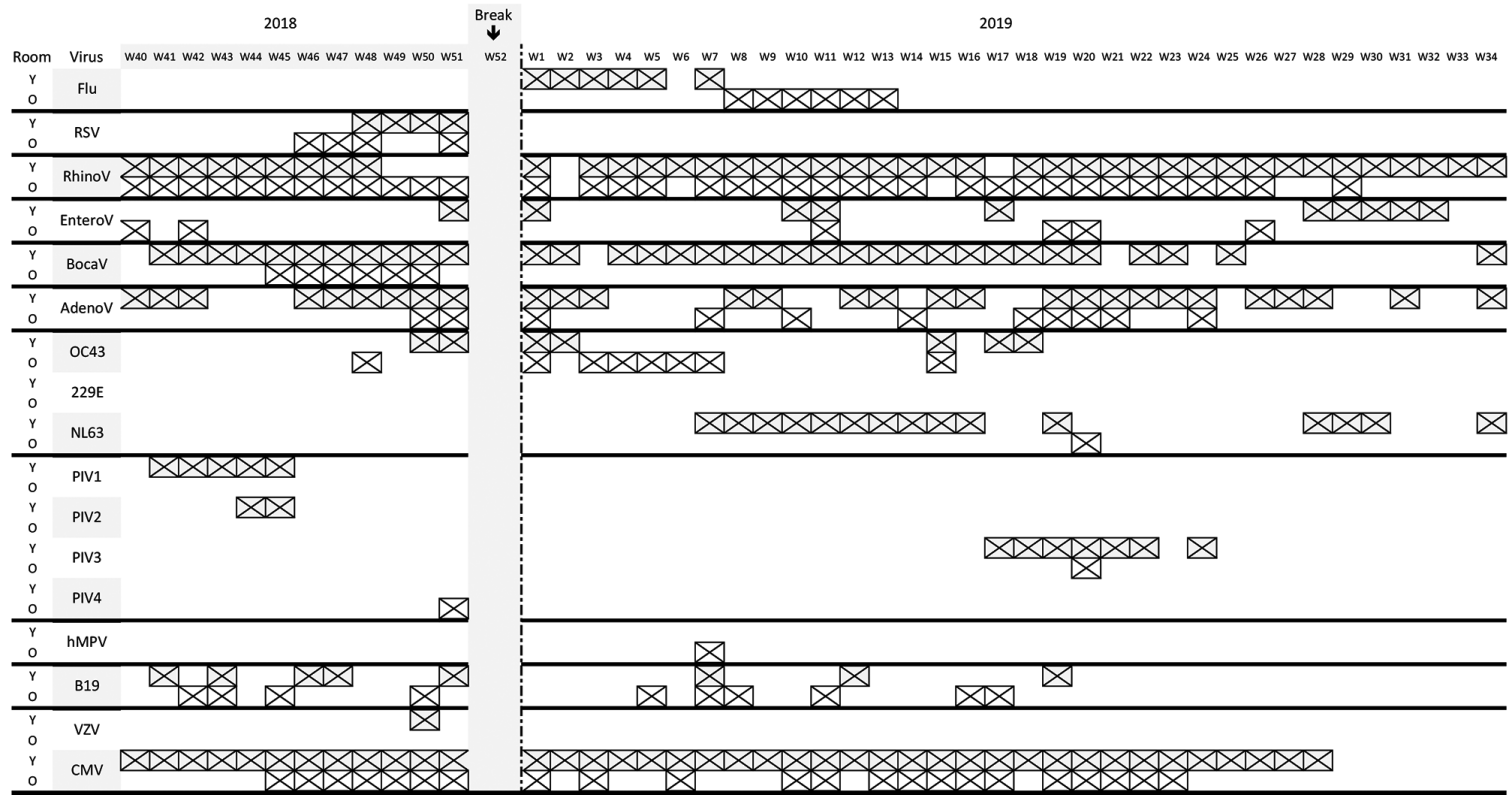
Detection of viruses by age group during study involving used facial tissues to monitor and diagnose viral respiratory infection. Each week is represented by a column, and detection of different viruses is indicated by a crossed cell. 229E, coronavirus 229E; OC43, coronavirus OC43; NL63, coronavirus NL63; AdenoV, adenovirus; BocaV, bocavirus; CMV, cytomegalovirus; EnteroV, enterovirus; Flu, influenza virus; hMPV, human metapneumovirus; O, older age group; PIV, parainfluenza virus; RSV, respiratory syncytial virus; RhinoV, rhinovirus; VZV, varicella zoster virus; W, week; Y, younger age group.

For the first study, used facial tissues were not collected when the daycare facility was closed during Christmas break. Rhinovirus and bocavirus were detected all year long, particularly from the youngest children. Other viruses were detected sporadically throughout the study period (Figure 1). Except for rhinovirus and bocavirus, viral infection in the youngest and oldest children was not synchronously detected. Influenza virus was detected as soon as week 1 in 2019, although the peak in the general population occurred only in week 6. In both age groups, parvovirus B19 was regularly but not continuously detected. By contrast, CMV detection was persistent among the youngest and intermittent among the oldest children.

**Figure 2:**
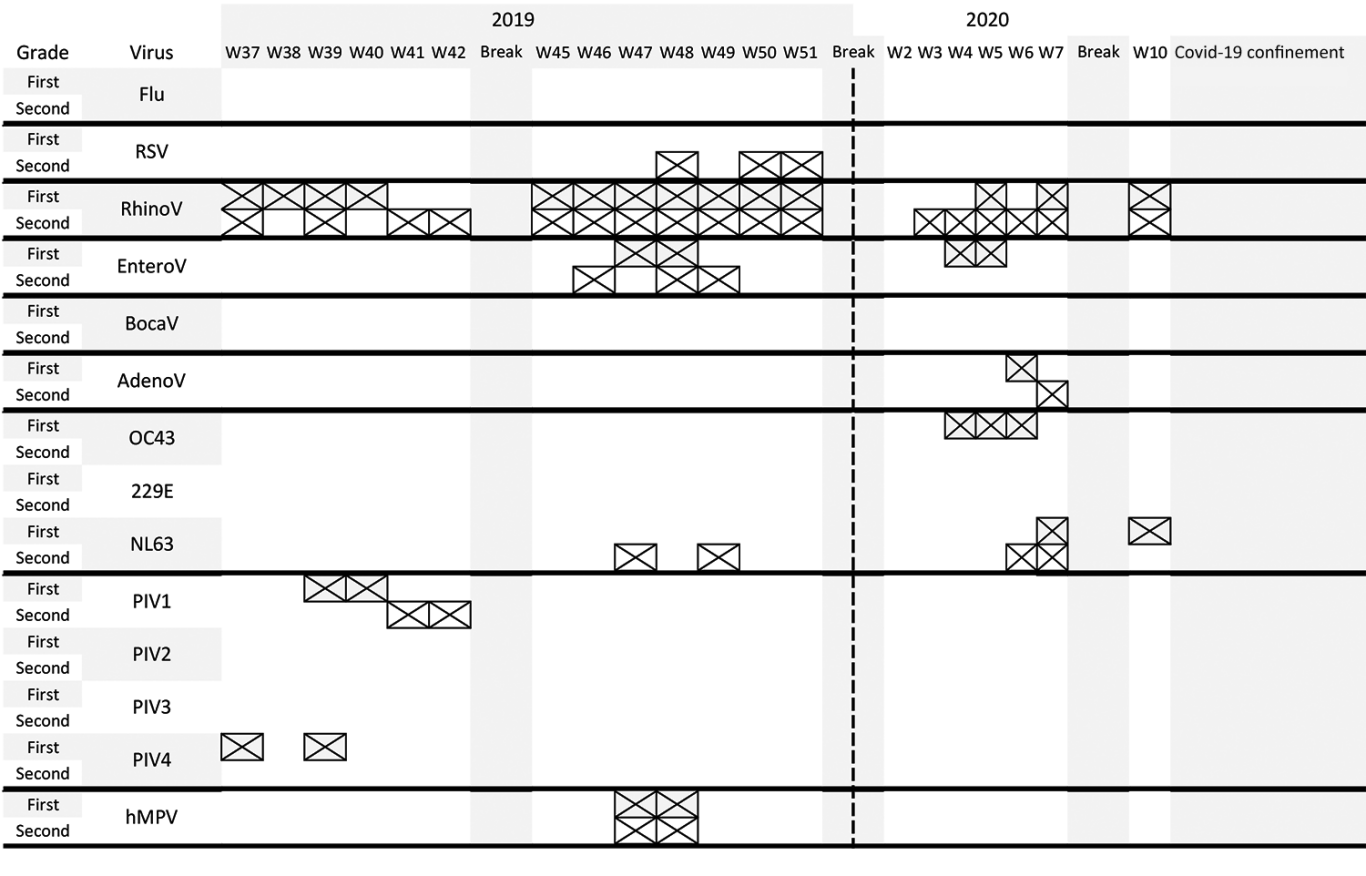
Detection of viruses by grade (first or second) during study of used facial tissues to monitor and diagnose viral respiratory infection. Each week is represented by a column, and detection of different viruses is indicated by a crossed cell. AdenoV, adenovirus; BocaV, bocavirus; EnteroV, enterovirus flu, influenza virus; hMPV, human metapneumovirus; PIV, parainfluenza virus; RSV, respiratory syncytial virus; RhinoV, rhinovirus; W, week.

The second study was unexpectedly interrupted by the confinement declared by the government of France after emergence of the COVID-19 pandemic. Rhinovirus was detected at almost all times, but other viruses were sporadically detected (Figure 2). Except for hMPV detected for children in both classes on weeks 47 and 48, viruses seemed to circulate independently among children in both classes.

### Individual Collections

After validating pooled collections from the daycare center, we proposed an individual approach to investigators and their relatives as soon as they exhibited influenza-like signs/symptoms. To process single facial tissues, we adapted the process to using a 60-mL syringe and a 7-mL volume of DPBS; after several steps of optimization, we retained these conditions. We sent 22 used tissues to the laboratory, where they were processed accordingly. All but 2 samples were positive for >1 virus. Viruses detected during the past 3 years were rhinovirus, bocavirus, influenza B virus, hMPV, coronaviruses 229E and OC43, adenovirus, and SARS-CoV-2. Some diagnoses were performed on samples that had been shipped through regular postal mailing. Most tissues were stored in standard plastic bags at room temperature for several days before being analyzed; there was no noticeable loss of sensitivity.

This in-progress work validates the feasibility of the approach on an individual basis. For most volunteers, tissue analysis enabled identification of the virus responsible for the observed signs/symptoms. As expected, all volunteers recovered from common RVIs within a few days.

**Figure 3:**
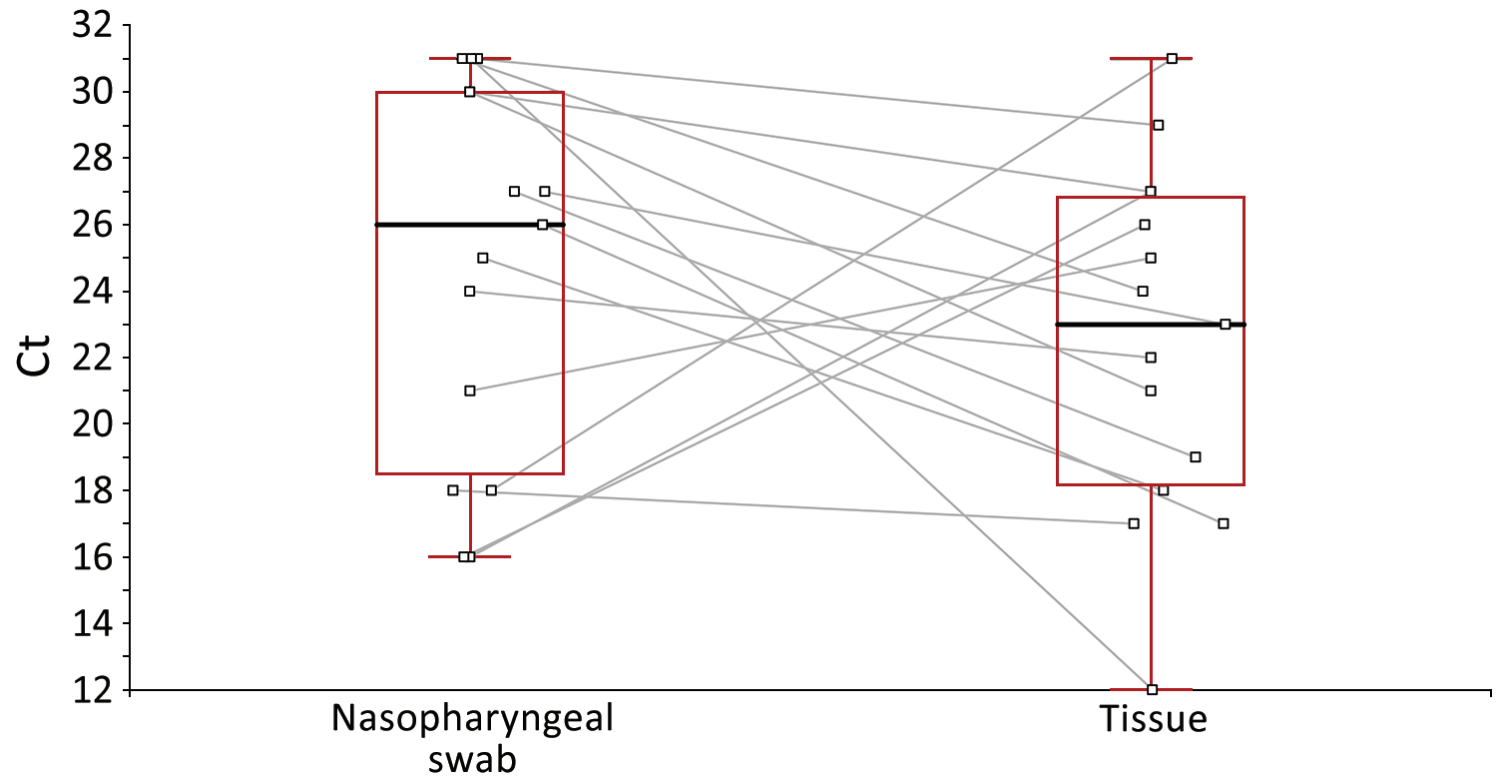
Reverse transcription PCR signal (Ct) obtained from nasopharyngeal swab samples collected from persons with COVID-19 compared with Ct obtained from processed used facial tissues in study of using facial tissues to monitor and diagnose viral respiratory infection. Each square indicates a patient, and observations for the same patient are linked between plots. Black lines within boxes indicate medians; box tops, 75th percentile; box bottoms, 25th percentile; and whiskers, maximums and minimums. Ct, cycle threshold.

When we compared signals obtained on both matrices collected from the 15 volunteers on the same day, median Ct was 23 on facial tissues but only reached a median Ct of 26 on nasopharyngeal swab specimens. The difference did not reach statistical significance (Figure 3), but Cts were lower for 11 of the 15 tissues than on swab samples. Values were within the typical standard range for SARS-CoV-2–infected patients.

**Figure 4:**
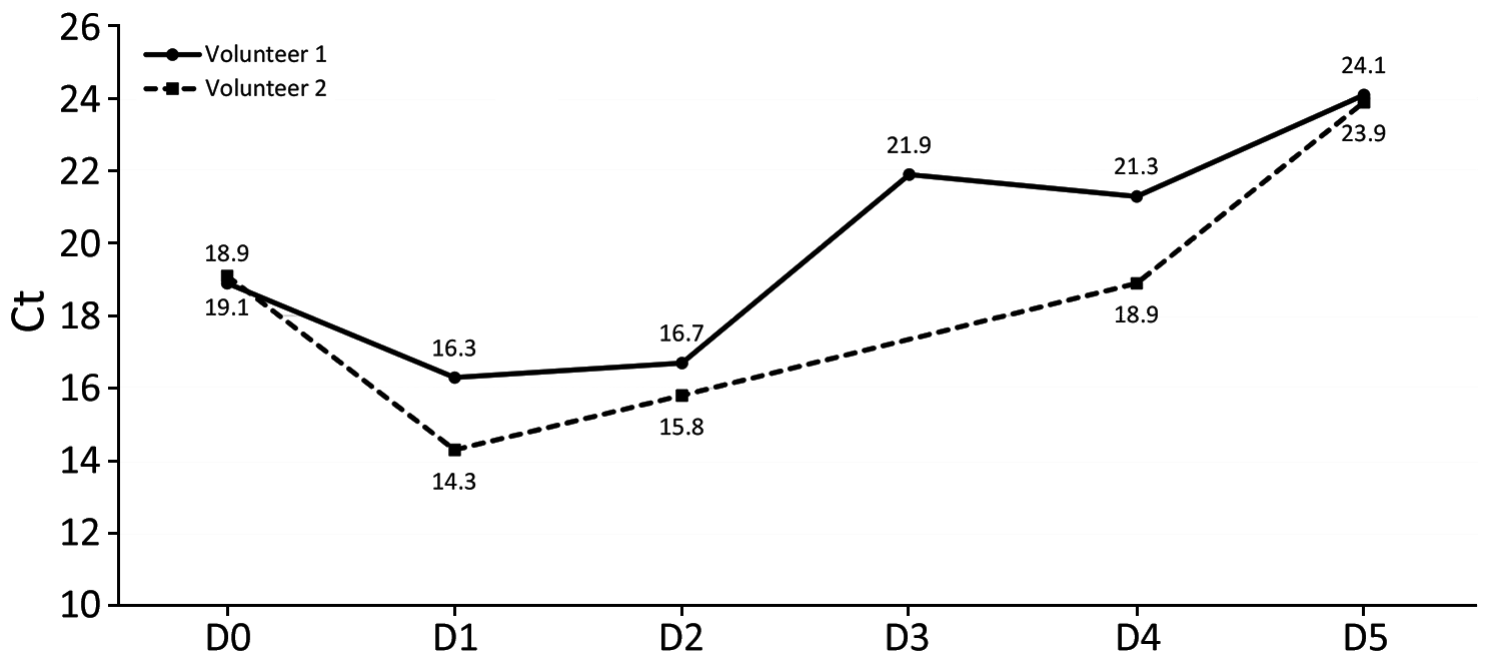
Ct values obtained from used facial tissues collected each morning after the first day from 2 volunteers in study of using facial tissues to monitor and diagnose viral respiratory infection. Volunteer 1 (closed circle, solid line) and volunteer 2 (closed square, dotted line) provided tissues every morning for 5 days (except day 3 for volunteer 2). Each tissue was analyzed as described, and SARS-CoV-2 RNA was detected using an Alinity assay (Abbott, https://www.corelaboratory.abbott). Day 0, the day of COVID-19 diagnosis performed on a typical nasal swab sample. Ct, cycle threshold.

For the 2 COVID-19–positive volunteers who provided daily facial tissue samples, the Ct obtained from tissues was lower than that from nasopharyngeal swab samples (19 vs. 27 and 23 vs. 27) (Figure 4). At each time point, the signal was positive; Ct ranged from 14 to 24. For 1 volunteer, at a later time point (11 days after initial diagnosis), Ct was 35.

## Discussion

We initially designed this study as a proof-of-concept study; our objective was to describe the circulation of respiratory viruses in communities of children. The results led us to consider a more individual approach, and we were able to gather enough data despite the COVID-19 pandemic. In the first part of the study, using pooled material, we gathered a large amount of data. Collecting used tissues from both institutions, the daycare center and the preschool, did not raise concerns from the professionals or the parents; both groups seemed interested in the study and awaited the weekly results. One objective of the regular monitoring of virus circulation would be to communicate the results on a weekly basis throughout the year to limit the unnecessary use of antimicrobial drugs when a viral infection is identified in a community, to inform the parents about what to do in such circumstances, and to possibly limit general practitioner visits. Parents who are informed about the circulation of a specific virus in a group of children may be less worried and could share their concerns with others. Collecting used tissues is noninvasive, well accepted by children and parents, and provides valuable information about the epidemiology of viruses in a specific community.

Despite proximity, older and younger children were not affected by the same viruses at the same time, which indicates that hygienic rules were probably well applied, particularly in the daycare center, but also that children of different ages are not susceptible to the same infections. That information reflects development of immunity during growth, but collecting data throughout the year demonstrates the susceptibility of children in each age group to viral infection.

Constant circulation of rhinovirus was not unexpected and may indicate rather weak immunity to the virus and highlights the relatively mild signs/symptoms in young children. Detecting parainfluenza virus in autumn in both communities of children fits well with the recent work from Horemheb-Rubio et al. (*14*). The start of the school year, when children gather, certainly plays a role in transmission of those viruses. Of note, parainfluenza virus 3 was also detected in the daycare center after the March break and persisted for several weeks.

Detecting CMV in the daycare center is informative and underlines the limits of our approach. Indeed, and particularly for CMV, detecting viral genome in pooled material may indicate either that the virus is perpetually circulating and propagated among children or that 1 or a few infants, those infected before or at birth, continuously shed virus (*15*). It is not possible to certify if detection comes from a long period of virus shedding, particularly if the level of secretion is high, as seen with CMV after maternofetal infection. For both community studies, we considered it unethical to perform individual analyses. Detecting 1 positive child would have led to possible discrimination or even eviction from the group.

In addition to the possible bias that detecting a virus in a pool of facial tissues may reflect only 1 long-shedding child, it is also possible that 1 child put >1 tissue in the collecting container. Those biases should be taken into consideration, and solutions should be sought to limit their influence in future similar studies.

Another objective of our community testing strategy would be to propose a surveillance model based on few strategically chosen groups. Seasonal virus infections propagate very efficiently among young children, and monitoring those communities may be useful for predicting emergence of winter seasonal viruses. In the study performed in the daycare center, the first influenza-positive collection was detected 6 weeks before the seasonal peak in the general population. Identifying such emergence early would leave enough time to deliver prevention messages (e.g., mask use) to the population. One may imagine a surveillance model similar to what has been done with sewage water for SARS-CoV-2 (*16*). Information from such surveillance would be communicated to the general population to better convey which preventive measures to take. Depending on the objective, several strategies could be considered. Because RVIs are probably transmitted by children, regular pooled tissue testing in a few representative classrooms would provide valuable information. Another approach would be to rely on a network of collaborative general pediatricians selected according to recruitment, who could collect tissues from children with respiratory signs/symptoms. Such surveillance requires individual participation, as opposed to wastewater testing, yet it may provide much more precise information about a specific population.

Individual testing was not a priority in our study, but detecting viral nucleic acid from numerous different viruses in tissues sent to our laboratory gave us confidence that that strategy could be pertinent. The COVID-19 epidemic gave us the opportunity to validate our approach. When tissues were collected from persons with COVID-19 diagnosed from standard nasopharyngeal swab samples, the signal obtained from tissues was higher in 73% (11/15) of cases (Figure 3). The median Ct value obtained from tissues was 3 units lower than that from classical nasal swab samples, a difference that was not statistically significant, possibly because of the low number of samples tested in parallel.

Blowing in a tissue is an individual action, and its effectiveness largely depends on the person. Nasopharyngeal swabbing also depends on the skill of a professional but may be less subject to variability. One possible way to assess the sampling quality is to quantify a cellular gene, yet this strategy is not routinely applied and lacks consensus among scientists (*17*). Quantifying a cellular gene on each tissue sample might validate the quality of the blowing. We anticipate that a threshold would have to be defined according to the tested population; elderly persons may not be able to blow as vigorously as younger persons. The kinetics performed on samples from the 2 COVID-19–positive volunteers who provided daily tissue samples demonstrate that SARS-CoV-2 nucleic acids are easily detectable on tissues for a long period. Those kinetics resemble kinetics usually described for large cohorts (*18*). The most sensitive approach might be having persons blow their nose in the morning because nasal fluids and dying cells accumulate in the respiratory tract during sleep. The influence of the timing for tissue collection should also be investigated. For the 2 COVID-19–positive volunteers, tissues were collected early in the morning at awakening, and neither person experienced any nasal congestion or rhinorrhea. Vigorous blowing was recommended to both participants. For those kinetics, but also for some individual diagnoses performed on demand, tissues were stored for several days at room temperature or even shipped by mail. Reverse transcription PCR performed on 32 tissues collected from volunteers positive for COVID-19, as documented by an antigen test and stored for a median of 12 days, were all positive, indicating that tissues can be stored and shipped without too much loss of sensitivity (data not shown). Possibly offering at-home sampling and remote diagnosis is a unique asset of this approach. Remotely testing patients should be considered when a viral infection emerges with signs/symptoms not requiring specific medical care. Such was the situation with COVID-19; many patients did not require medical attention. Leaving patients the possibility of collecting the sample themselves without being in contact with medical staff or other persons would limit the risk of propagating any emergent virus. Although the best matrix for performing COVID-19 diagnosis, has been debated, a unique tissue can be loaded first with nasal discharge and then sputum for 1 patient, which may increase sensitivity when nasal discharge is limited (*7*). The possibility of combining 2 matrices on the same sample may increase clinical sensitivity of the used-tissue strategy. Few of the tested tissues had both materials, but we cannot affirm that the performance was improved by such an approach. Because this method was developed to speed access to diagnosis, we made no recommendations regarding the brand or the kind of facial tissue to use. A formal assessment of our method on different tissues is required, yet common sense indicates use of commonly available commercial unperfumed plain white facial tissue.

Of note, >1 sample positive for >1 virus was analyzed on the different systems routinely used in our laboratory. A valid result was obtained on all platforms used. Those results probably reflect improved extraction processes for most commercially available automated systems. In particular, no PCR inhibition was observed for the analyzed tissue liquids. Moreover, complete sequences of SARS-CoV-2 can also be obtained from tissues; an example of 1 sequence obtained from used tissue is referenced in GISAID (https://www.gisaid.org; identification no. EPI_ISL_13050738).

This study reflects major changes in virology that have occurred in recent years. Although growing the virus in cell culture was the reference for diagnosis, we slightly moved to molecular detection of viral nucleic acids. The information gained by each strategy is different: in the first instance, we identified live virus, probably infectious; in the second, we found only the presence of viral components. Yet the overall sensitivity of nucleic acid amplification methods now offers improved clinical sensitivity despite loss of information regarding the viability of the virus and possibly its infectiousness. Detecting viral nucleic acids in used tissues is feasible and possibly as sensitive as classical sampling methods, if not more so. That possibility provides the option for virus testing of pooled samples in communities to provide information on the dissemination of any respiratory-transmitted virus and to propose a noninvasive method for the general population. Situations in which persons need to be repeatedly tested, such as athletes during a several-day event, may also largely benefit from this strategy. Being able to send tissues by regular mail for remote diagnosis is also a real asset with potential application for gaining information about the diffusion of an infection in a population. Although this study should be considered as a proof-of-concept work, we believe that the accumulation of data justifies considering the use of our methods on a larger scale.
